# What Is Inflammatory Breast Cancer? Revisiting the Case Definition

**DOI:** 10.3390/cancers2010143

**Published:** 2010-03-03

**Authors:** Paul H. Levine, Ladan Zolfaghari, Heather Young, Muhannad Hafi, Timothy Cannon, Chitra Ganesan, Carmela Veneroso, Rachel Brem, Mark Sherman

**Affiliations:** 1 The Department of Epidemiology and Biostatistics, George Washington University School of Public Health and Health Services, Washington, DC, 20037, USA; E-Mails: ladan_z@yahoo.com (L.Z.); bschay@gwumc.edu (H.Y.); mhafi@msn.com (M.H.); timmyut21@hotmail.com (T.C.); cganesan@gwu.edu (C.G.); cveneroso@juno.com (C.V.); 2 The Department of Radiology, Medical Faculty Associates, The George Washington University Medical Center, Washington, DC, 20037, USA; E-Mail: rbrem@mfa.gwu.edu; 3 Division of Cancer Epidemiology and Genetics, the National Cancer Institute, Bethesda, MD, 20892, USA; E-Mail: shermanm@mail.nih.gov

**Keywords:** inflammatory breast cancer, AJCC, SEER, diagnosis, biology, clinical presentation

## Abstract

The case definition for inflammatory breast cancer (IBC) is controversial. The American Joint Committee on Cancer defines IBC as redness, warmth and edema involving at least half the breast. The SEER program relies on a pathologic finding of dermal lymphatic invasion and recently added those with clinical involvement of more than 3/4 of the breast. We established a registry to collect information and specimens from IBC patients to clarify the epidemiology and biology of these tumors. The goals of this report are to suggest improvements regarding case definitions and provide data on the variety of presentations relevant to early diagnosis.

## 1. Introduction

Inflammatory breast cancer (IBC) is an aggressive form of breast cancer that disproportionately affects women who are young or African-American [1,2]. Although all studies agree that IBC is a biologically virulent type of breast cancer, there is considerable debate about whether the diagnosis of IBC should be based on clinical presentation, histopathologic features, or some combination. The American Joint Committee on Cancer (AJCC) criteria for diagnosis is based on clinical features rather than pathologic ones [3]. In the past, in contrast, the National Cancer Institute’s Surveillance Epidemiology and End Results (SEER) Program has emphasized histopathologic demonstration of invasion of dermal lymphatics as a diagnostic criterion, although more recently, clinical criteria have been added [2]. The application of non-uniform criteria for the diagnosis of IBC by different organizations, clinicians and researchers has impeded efforts to define the biology and clinical behavior of this entity.

To address uncertainties about the diagnosis and pathogenesis of IBC, we have developed a United States and Canadian Inflammatory Breast Cancer Registry (IBCR). Our growing experience with the IBCR is showing the difficulties in making the diagnosis of IBC in community practice and in research publications. In this report, we describe competing and conflicting approaches for the diagnosis of IBC, highlight the implications of varying diagnostic criteria for interpreting the literature, and outline our efforts to improve the recognition of this entity. We attempted to preliminarily assess whether candidate IBC cases, sub-classified by clinical and/or pathologic features, shared similar laboratory characteristics and outcomes.

## 2. Results and Discussion

### 2.1. Results

There are 50 patients in this study. The geographic distribution of these patients included 22 U.S. states, the District of Columbia, and 2 provinces in Canada. A total of 42 (84%) subjects contacted us because of two internet web sites; the other 8 patients were from the metropolitan Washington, D.C. area with 7 of them treated at The George Washington University Medical Center. The patients included 49 Caucasian women and one African American with an overall mean age of 49 years (range = 30 to 83 years). One 16-year old girl, who did not meet the minimum age requirement for participation of 18 years, was excluded, and died before she was 18.

**Figure 1:**
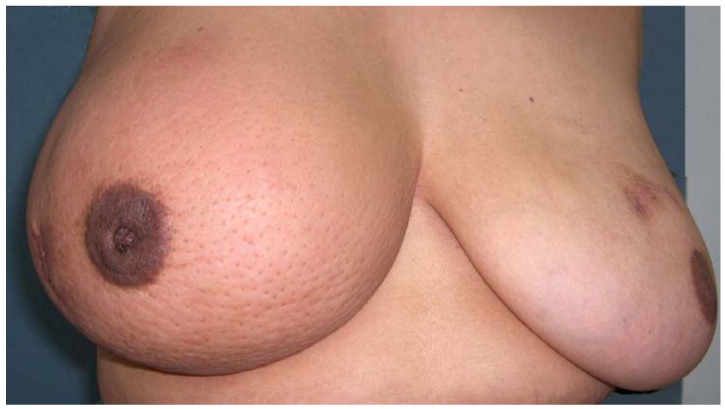
Clinical photo of IBC patient. Inflammatory breast cancer of the right breast characterized by marked edema and swelling, erythema and dimpling ("peau d'orange") characteristic of the patients meeting the AJCC case definition. Photograph provided from study of Inflammatory Breast Cancer funded by the Intramural Research Program of the Division of Cancer Epidemiology and Genetics, National Cancer Institute, National Institutes of Health, Department of Health and Human Services.

Patients were divided into six diagnostic categories on the basis of clinical and pathological features (Table 1). Patients in Category 1 were those who met all AJCC and SEER criteria for the diagnosis of IBC. The AJCC states “inflammatory carcinoma is a clinicopathologic entity characterized by diffuse erythema and edema (peau d’orange) of the breast, often without an underlying palpable mass. These clinical findings should involve the majority of the skin of the breast” [3]. (See Figure 1) The SEER criteria initially required pathological confirmation (dermal lymphatic invasion or DLI) coded as ICD-0-2-8530-3, but recent modifications allow use of clinical codes in cases lacking diagnostic histopathologic criteria but with marked involvement of the breast, specifically Extent of Disease (EOD)-S 998, “diffuse; widespread: ¾ or more of breast” [2]. Those in Category 2 met AJCC criteria but did not have documented DLI. Those in Category 3 would have been classified as IBC by SEER since they were pathologically confirmed with DLI but would not have met AJCC criteria for the diagnosis. *i.e*., less than half of the breast was involved. Category 4 would not have been classified as IBC by either AJCC or SEER but were designated IBC by practicing physicians because the clinical findings of IBC involved less than half of the breast and DLI was not observed. Category 5 with only DLI findings meets the criteria of “occult IBC” described by Saltstein [4] and Category 6, designated “IBC features in recurrent breast cancer,” attempted to include those case designated by Taylor and Meltzer [5] as secondary IBC. 

**Table 1:** IBC Categories.

Category	Description	N	Percent
Category 1	Classical history and physical findings, pathological confirmation (invasion of dermal lymphatics)	16	32
Category 2	Classical history and physical findings, no pathological confirmation	6	12
Category 3	Clinical findings involving less than half the breast, pathological confirmation	8	16
Category 4	Clinical findings involving less than half the breast, no pathological confirmation	15	30
Category 5	Pathologic findings without clinical features (“occult IBC”)	1	2
Category 6	IBC features in recurrent breast cancer (“secondary IBC”)	4	8

Significant redness of the skin, sometimes described as a rash, was the most frequent initial symptom (25, 50%); breast enlargement (22, 44%) and pain (17, 34%) were also prominent symptoms (Table 2). Severe pruritus was reported by 8 women (16%) as the predominant initial symptom. A discrete, palpable mass was present in 11 women (22%) with an inverted nipple also being the initial symptom in 11 women (22%), these findings based on self-examination. 

**Table 2:** Presenting Symptoms.

Symptom	Number	Percent
Redness/Rash	25	50%
Enlargement	22	44%
Pain	17	34%
Discrete Lump	11	22%
Inverted nipple	11	22%
Itching	8	16%
Peau d’orange	7	14%
Warmth	6	12%
Thick mass	3	6%
Other	12	24%

Initial clinical diagnoses for 10 women were infection, including nine diagnoses of mastitis and one of cellulitis (Table 3). These 10 women were treated with antibiotic therapy for up to 5 months prior to a definitive diagnosis of breast cancer. Cancer was suspected in 35 (70%) patients but a brief trial of antibiotics was attempted prior to cancer work-up in 6 of these patients (12%). The work-up for breast cancer began immediately in 29 (58%) patients after first medical contact.

**Table 3:** Type of Initial Diagnosis for Cancer.

Initial Diagnosis	Number	Percent
Breast Cancer	29	58%
Mastitis	9	18%
Breast Cancer *vs*. Mastitis	6	12%
Cyst	1	2%
Ductal Papilloma	1	2%
Nothing to worry about	1	2%
Fibrous mass	1	2%
Cellulitis	1	2%
Hematoma secondary to trauma	1	2%

**Figure 2:**
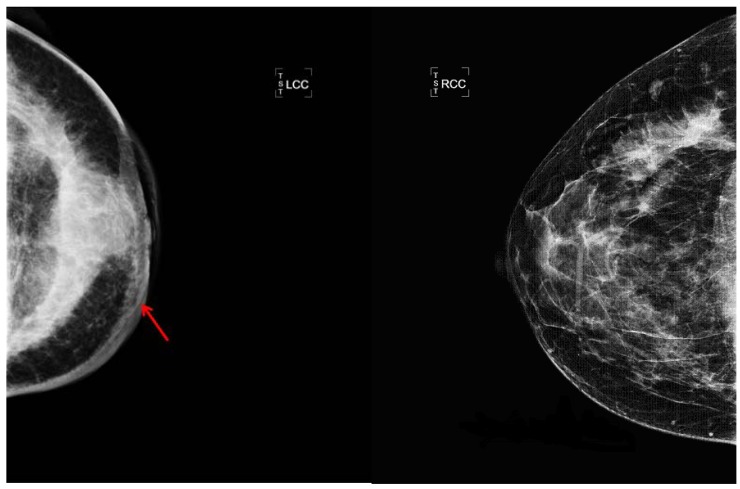
Radiologic photos of IBC patient. The arrow shows thickening of the skin of the left breast due to edema which is characteristic of inflammatory breast cancer. The density in IBC cases may be diffuse or localized. The other mammogram is of the uninvolved right breast.

A discrete mass and thickening of the skin was the most consistent mammographic findings (Figure 2): a discrete mass was seen in 14/48 (29%) of evaluable patients and skin thickening was documented in 13 (27%). Thirty three women had sonographic examination in addition to mammography; six were concordant with mammograms revealing a discrete mass and six showed a mass on sonogram not apparent on mammography. Only one patient had a mass identified on mammogram but not on sonogram. Eighteen patients (37.5%) had no masses detectable by either mammogram or sonogram. Enlarged lymph nodes were recognized radiologically in 8 patients and asymmetric densities were also recognized in 8. 

Forty-seven (94%) of the women in this study had infiltrating ductal carcinoma and one had lobular carcinoma. One patient with predominantly ductal features in the primary tumor had lobular carcinoma in the metastases and one patient had a mucinous cancer. The majority of patients (27/49, 55%) were estrogen receptor positive and 28/49 (57%) progesterone receptor positive. Less than half (16/43, 37%) were Her-2 neu positive. In the 25 patients enrolled within 18 months of diagnosis, the three year survival was similar among ER positive patients, 11/16 (69%) *vs.* 6/9 (67%) in ER negative patients. Patients positive for Her2-neu (4/5) had an 80% three year survival *vs*. 67% in Her2-neu negative patients (8/12). 

As far as pathological findings, 22 (44%) patients were noted to have DLI. 16 (32%) were specified as having no DLI, and there was no mention of the presence or absence of DLI in 12 (24%) patient reports. Tissue samples were available from 29 of the patients to be examined by one pathologist. The samples were either from FNA, lumpectomy, excisional biopsy, or mastectomy. Of these, all 29 were infiltrating ductal carcinoma and 28 of 29 were grade III while one was grade II. DLI was noted in 10 of the patients, was absent in 9, and the sample was insufficient in 10. One of the patients with DLI was lost to follow up.

Most patients 43 (86%) received neo-adjuvant chemotherapy for treatment. Six patients were not diagnosed initially as having IBC and were treated with surgery for their breast cancer (this included one patient with secondary IBC whose inflammatory signs only appeared with recurrence). One patient refused chemotherapy because she had multiple sclerosis and decided she would restrict her treatment to radiotherapy. Of the 43 evaluable patients with neoadjuvant chemotherapy, 13 had a complete clinical response, 25 had a partial response, and 5 had either minimal or no response. The pathologic response to chemotherapy was less impressive than the clinical response, with only two patients obtaining complete pathologic response (pCR). The majority (47/50, 94%) of patients had a mastectomy; two decided that their disease was already too far advanced and one declined for religious reasons. 45/50 (90%) had radiation therapy, usually following mastectomy. 

We compared several cell surface markers by our Categories, comparing patients who met AJCC criteria (Categories 1 and 2 of which there were 22 patients) with those that did not (Categories 3 and 4, of which there were 23 patients). The percentage of patients meeting AJCC criteria who were positive for estrogen receptor, progesterone receptor and Her2Neu markers were 59% (13/22), 45% (10/22) and 32% (7/22) respectively; these percentages were similar to those not meeting AJCC criteria that had rates of 52% (12/23), 39% (9/23) and 30% (7/23) respectively. There was also no significant difference in survival by Category. Follow up information was available for all patients except one (for whom there has been no follow up since 2004). The median progression free survival (PFS) was similar for those meeting AJCC criteria compared to those not meeting AJCC criteria at 110 weeks, and the three year survival rate for women who were enrolled within 18 months of diagnosis was 75% among those meeting AJCC criteria (9/12) and 73% among those not meeting AJCC criteria (8/11).

### 2.2. Discussion

The understanding of the etiology and biology of IBC is greatly hampered by the multitude of definitions and names that have been applied to this disease. These conflicting case definitions have impeded attempts to review the effect of different therapeutic approaches to IBC [6]. An example of the importance of clarifying the case definition is that according to SEER data, the incidence of IBC is increasing while the incidence of breast cancer in general is not [2,7]. We recently reviewed the multitude of case definitions, including pseudo-IBC, occult IBC and secondary IBC [8], and the data suggest that categorizing cases by the extent of clinical and pathologic findings is more productive than attaching labels to groups that are not readily reproducible. The development of the Categories that we used for the IBCR had its basis in our earlier studies in Tunisia [9,10] which were based on the original observation that all three forms of rapidly progressing breast cancer or pousee evolutive, which included a category PEV 3 which met the AJCC criteria for IBC, had a similarly poor prognosis compared to breast cancer cases with less dramatic skin involvement (PEV2 which was defined as involving less than half the breast) [11]. Focusing on the clinical features, Categories 1 and 2 included patients with clinical involvement of more than half of the breast. These patients meet the criteria required by AJCC for the diagnosis of IBC and by the Institut Salah Azaiz in Tunisia for PEV 3. PEV 2, which was defined in Tunisia as “a tumor whose overlying breast tissue, skin in particular, was affected by subacute inflammation and edema involving less than half of the breast surface,” was used as our case definition for Categories 3 and 4. We did not consider PEV 1 (rapid growth without external skin manifestations) and Category 5 (“occult IBC”) in this analysis because neither had clinical manifestations of IBC. In regard to Category 5, three studies have shown that the outcome in this group is significantly better than cases with clinical involvement of the skin [12,13,14]; therefore “occult IBC” is more appropriately considered within the spectrum of non-inflammatory aggressive IBC. 

All of the analyses in this study indicate that those cases with the clinical features of IBC, but not meeting AJCC criteria, do not differ from those that do meet AJCC criteria, with hormone receptor status and the presence of Her2-Neu markers being similar in both groups. Regarding clinical outcomes, PFS and the three year survival rate in those meeting AJCC criteria (Categories 1 and 2) were no worse than in those not meeting these criteria (Categories 3 and 4). This similar three-year survival rate in cases meeting and not meeting the AJCC case definition supports the contention [1,2] that the AJCC case definition misses a large number of IBC cases and that the ominous significance of even limited clinical evidence (redness, warmth, edema, sudden pain) are sufficient signs of IBC to warrant immediate neoadjuvant chemotherapy as well as the aggressive complementary measures offered to those with more extensive clinical manifestations. While the three year survival rate is greater than 50% in this series, this is reflective of the improvement in IBC survival over the years and does not indicate any bias towards survival in case ascertainment as it included only patients who were entered within 18 months of diagnosis, where there is a negligible mortality from IBC [1,2]. 

Although the IBCR is not a population-based registry, this preliminary descriptive analysis has revealed findings related to age, hormone receptor expression, and poor prognosis consistent with studies based on population-based data [1,2,7,15]. It should be noted that although the percentage of ER+ women is less than for non-IBC breast cancer patients, the general statement that IBC is usually ER- does not appear to be correct. The finding of 55.5% (25/45) women in our registry who are ER+ is similar to the 54.3% (1264/2329) in population-based studies [2]. One difference, however, between the population in this study and the population in the SEER studies [1,2,7] is that our population had a smaller African-American population: 2% in our study *vs*. 14% in the latest SEER study [2]. This difference is probably due to the fact that most of our patients came though the internet. In our study, we have found that the prognosis of women in the IBCR was poor, regardless of whether patients met AJCC or SEER criteria as previously reported. The progression-free survival in those meeting AJCC criteria (Categories 1 and 2) was similar to those not meeting AJCC criteria (Categories 3–4). 

Our observations suggest that patients need to know the importance of seeing breast specialists early because many doctors are inexperienced with IBC and consequently there may be a prolonged delay. Many doctors mistake the signs of IBC for an infection. Additionally many believe that a painful breast in a young woman “can’t be cancer.” A high index of suspicion should be maintained since the diagnosis in a 16 year old in the United States reported to our Registry is not unique [16]. Our data emphasize early focus on IBC even if less than half of the breast is affected. Regarding differentiating between mastitis and IBC, it is reasonable to try a short course of antibiotics since it may be inadvisable to biopsy an infectious process, but clinically, it is important to note that mastitis is generally seen in association with a recent pregnancy and is almost never seen in a post-menopausal woman. Reliance on mammography for differential diagnosis could be very misleading since most cases of IBC do not show the same discrete lump usually seen with non-IBC breast cancer. Another important clinical consideration is that while the prognosis in IBC is not as favorable as in breast cancer in general, the prognosis is improving [2]. 

Clinically it should be noted that reliance on mammography for the diagnosis of breast cancer in a patient with IBC requires a high index of suspicion since a discrete mass is usually not identified. More typically, secondary signs of breast cancer are present such as skin thickening and prominence of the breast trabeculae and diffuse increased density. Since mammography cannot differentiate IBC from mastitis, the reliance on additional imaging modalities such as MRI can be useful. MRI can help identify a mass which may be obscured by the increased density seen with mammography. Furthermore, MRI is a more accurate imaging approach to assess pectoral muscle involvement and may be extremely helpful in assessing response to neoadjuvant chemotherapy. Regarding therapy, the standard procedure has been well described by Bristol *et al*. [17] and consists of chemotherapy, surgery, more chemotherapy and radiation therapy. Forty-four of our patients followed this general protocol. 

## 3. Experimental

The IBCR was established June 1, 2002 to collect standardized information from IBC patients in the U.S. and Canada. Patients with IBC were entered into the Registry if they agreed to be interviewed by us relating to the clinical presentation of their disease and to their exposure to certain breast cancer risk factors and if they agreed to give us authorization to access their medical records and pathologic specimens. All patients signed an Informed Consent. This report summarizes the results from the first 50 patients registered.

The patients in this study were all women at least 18 years of age who contacted us after learning about the Registry on the internet or from local oncologists and who agreed to be interviewed and to provide access to medical records and tissue blocks. Their entry into the Registry varied between less than three months to more than two years after diagnosis. Survival data was used for those women enrolled within 18 months of diagnosis. All women had two interviews, the first by one of us focusing on the clinical history, family history and any issues the women wanted to discuss concerning their illness; the second by a trained interviewer who asked prepared questions from a standardized form about clinical details and specific risk factors associated with aggressive breast cancer reported in earlier studies [18]. Most interviews were telephone interviews with the exception of 8 patients from the Washington, D.C. area who were interviewed in person. Hospital and out-patient records were obtained on each patient and served as the basis for specific diagnostic evaluation. These records included screening tests and pathology reports. Reports on pathology were reviewed by our investigators; all reports and tissue samples from 29/50 patients were reviewed by one pathologist. Mammogram and ultrasound reports obtained at the time of diagnosis were reviewed by one radiologist on all patients. 

The initial response to adjuvant chemotherapy was assessed by patient interview and medical records review. The response was determined clinically and pathologically. Clinically, the response was designated as complete if there was a complete response with resolution of all signs of tumor (redness, mass, peau d’orange) and the breast returned to normal; and partial if there was a definite response but residual breast abnormality, often with residual palpable tumor. The patients who did not meet the above criteria were considered to have no response. Pathologically, the patients were divided into a complete pathologic response or incomplete response with residual tumor in the mastectomy specimen, usually accompanied by persistent lymphadenopathy. Patients who had mastectomy prior to chemotherapy or no mastectomy were not evaluable. 

## 4. Conclusions

While the characterization of IBC utilizing the IBCR categories provides a useful approach to an important disease, a more accurate and useful test in the standardization of the diagnosis may be provided by laboratory assays. A number of approaches, such as that described by Houchens and Merajver [19], are of great importance not only because of their potential to improve the diagnostic specificity for epidemiological studies but also the potential to lead to improved modes of treatment. It is important to realize that while IBC is relatively rare, the numbers approximate or exceed those of many other malignancies. Our review of data from the SEER Program [2] demonstrates that IBC constitutes approximately 2.5% of all breast cancer cases. Data from the American Cancer Society [20] indicate approximately 192,370 new cases of invasive breast cancer in the U.S. in 2009, which would result in 4,810 new cases of IBC. This approximates the 5,760 estimated for acute lymphocytic leukemia and the 5,050 cases estimated for chronic myeloid leukemia for males and females combined in 2009 and is twice the number of cases in women alone, 2,410 and 2,120 respectively.
